# The requirements of cryptocurrency for money, an Islamic view

**DOI:** 10.1016/j.heliyon.2020.e03235

**Published:** 2020-01-16

**Authors:** Dodik Siswantoro, Rangga Handika, Aria Farah Mita

**Affiliations:** a Universitas Indonesia, Indonesia; b Institute for International Strategy, Tokyo International University, Japan

**Keywords:** Cryptocurrency, Money, Islamic, Paper, Gold, Coin, Financial economics, Financial market, International finance, Public finance, Money, Religion, Economics

## Abstract

This research aims to evaluate the suitability of cryptocurrency as money from the Islamic perspective. Money, in the Islamic perspective, has specific characteristics and requirements, such as stability and is based on assets. Cryptocurrency may not fulfil this as it has queries as money from the Islamic perspective. The research method applied data of 23 cryptocurrency prices and related information. The result shows that cryptocurrency is hugely volatile and has limits to being called ‘money,’ as it is limited and used for speculation, which is prohibited in Islam. The research implies that Muslims would be reluctant to use cryptocurrency as money, as a currency of transaction. This reason raise an expectation that the cryptocurrency will not develop rapidly in Muslim countries.

## 1 Introduction

The cryptocurrency was developed to facilitate peer-to-peer transactions that operate independently from the central bank. Cryptocurrency is not physical; it can be a coin but it is a digital coin. It also cannot be withdrawn. The owner can only transfer the funds to other parties. The application of cryptocurrency is an interesting issue of money and contributes to the financial market as it is based on a blockchain that is out of the current modern financial system of fiat money. Meanwhile, fiat money is produced by the central bank of each country; however, cryptocurrency is based on a borderless system as it is not limited to one country or territory. No authority controls the money mechanism of cryptocurrency.

The value of cryptocurrency is based on the blockchain creation to store the data. The calculation is based on the algorithm, which is complicated. The bigger the blockchain can create the data and system, the higher the fee of miners. This is the mechanism of how the value of cryptocurrency is created. Then, the supply and demand of users can also lead to either the increase or decrease of the value of cryptocurrency.

The issue of cryptocurrency from the Islamic perspective has been discussed. However, recommendations on this issue are absent. Most previous research is based on a qualitative study, which refers to classical Islamic law. There are pros and cons arguments for this. This gap would be proved by empirical data in this research. In the age of Prophet Muhammad PBUH, money was based on bullion gold and silver with specific characteristics. Money based on camel leather is temporarily permissible as a result of limited resources. Money today is more of a medium of exchange for goods and services that can be used by common people. Most Islamic scholars may propose fiat money for a medium of exchange only. This is the motivation of the research as money in the Islamic perspective discursion can be flexibly arranged in specific conditions and times. The motives for holding money, which is suitable to Islamic teaching, are (a) the transaction motive and (b) the precautionary motive. However, the speculation motive, or trading, is strictly prohibited (Sanusi, 2002) as this activity can be categorized as hoarding, which is prohibited in Islam. In addition, money should be treated as a currency of transaction rather than for gaining profit from speculation activity. This concept would be the basic requirement of the money characteristic from the Islamic perspective. Different with most of previous studies, this research will also discuss and provide analyse based on quantitative data.

The issue of cryptocurrency that can be functioned as money was discussed by Ammous (2018). He stated that the indicator of exchange can function as money as a store of value like Bitcoin. This is also supported by other factors, such as low growth supply, reliable network, and not much interference, which may not be found by other cryptocurrencies. Mills and Nower (2019) found that most traders faced high risk, but low depression, and anxiety concerning cryptocurrency trading. This is experimental to gamblers. Other researchers found that there were no herding activities in cryptocurrency trading as a trader may have an individual motive for cryptocurrency trading (Stavros and Vassilios, 2019). Meanwhile, Bouri et al. (2019) found a tendency of herding when uncertainty increased, which may cause cartel trading.

This research aims to identify if cryptocurrency fulfills money requirements. Money, in the Islamic perspective, historically should be based on physical assets, should be able to cover all activities, and is not for speculation purposes. The characteristics of cryptocurrencies will be analyzed based on money from the Islamic perspective in the current context. In fact, the percentage of the Muslim population in the world was around 24% in 2015 (www.bbc.com). This would affect the development of cryptocurrencies in Muslim countries if it is labeled as a prohibited activity or otherwise. Nevertheless, the development of cryptocurrencies has not been fast as it contains risks that can occur. This can be a consideration for Muslim countries to not apply it. In addition, in China and Russia, cryptocurrency is banned for similar reasons (www.investopedia.com).

The remainder of the paper is organized as follows. The second section discusses the literature review and the theory of money from the Islamic perspective and cryptocurrency study. The third section describes data and the adopted methodology. The fourth section analyzes the results. The last section concludes the paper. This may be the first research paper that discusses and promotes the legality of cryptocurrency in the Islamic perspective based on quantitative data. In addition, there are 23 cryptocurrencies that will be used for the analysis. So far, previous researches only analyzed less than five cryptocurrencies in the paper.

## 2 Literature review

### 2.1 History of money: the Islamic perspective

The history of money started when the Islamic government ruled. It started when Prophet Muhammad PBUH ruled in the city of Medina in 625 AD. The money was called the dinar, which was made of 4.25 g of 22-carat gold and dirham, which was made of 3-grams of silver. Previously, money was made of the skin of a camel, but then it was prohibited, as it can significantly reduce the camel population (Haneef and Barakat, 2002).

The dinar was actually copied from the golden money of the Byzantine Empire while the silver was copied from the Persian Empire. These two types of money had a stable ratio of 1:10 but then fluctuated to 1:15 in the long period (Rashid et al., 2002). Such a fluctuation occurred because of the hoarding by some people. In other words, they minted the dinar to gold because the dinar was made of gold, which was the intrinsic value of the dinar. This phenomenon was observed by Al Maqrizi. Then, the terminology known as bad money drove out good money by Gresham's law (Rosly and Barakat, 2002).

From the Islamic perspective, money is exclusively used for an exchange not for speculation or trading to gain profit purposely. Taking profit from money trading on purpose can be categorized as usury (riba) (Sanusi, 2002). On the other hand, referring to the phenomenon above, people would keep “good” money rather than to use for transaction what is “bad” money.

### 2.2 Mechanism of cryptocurrency

A cryptocurrency is a virtual coin so it does not have a physical form. The only proof of ownership of cryptocurrency is a recorded transaction on the blockchain. The blockchain is a public record (or electronic ledger). People who own cryptocurrency, for example, want to buy goods from sellers willing to accept cryptocurrency as payment. Rather than having a bank facilitate the transfer of the currency, that transfer takes place through the public ledger system.

The initiation of a new cryptocurrency usually arises when a company plans to launch new products and find funds to develop them. The company creates its own virtual currency and issues new coins or tokens created through the initial coin offering. Different labels have been used to describe various types of tokens or coins issued. The first cryptocurrency was Bitcoin. Bitcoin has no function or purpose other than as a means of exchange (or store of value).

Cryptocurrency is usually arranged by a set protocol that determines how many coins can be made, how they are made and how the integrity of ledgers is protected. These protocols are intended to be equivalent to government regulations and laws that support fiat money, and their strength will affect confidence in the digital currency and, therefore, their supply and demand. Usually, cryptocurrency blockchains are arranged so that it is difficult, or impossible, to change their operating protocol.

When a cryptocurrency enters circulation, there are many ways to get it. It can be purchased through exchanges; some people accept it because their company allows them to choose to receive digital money; some retailers accept cryptocurrency as payment for their goods and services; or people, or organizations, who maintain the blockchain for cryptocurrency are usually rewarded with cryptocurrency.

### 2.3 Cryptocurrency in the Islamic perspective

The issue of cryptocurrency as money is an interesting issue. This is because cryptocurrency was based on cryptography in the financial system. The value calculation of cryptocurrency is also based on an algorithm in the system of the blockchain. The blockchain is claimed to have some advantages as it is a safe, undeleted, complicated, and efficient system. To meet the criteria of a tool of exchange or money in Islamic teaching, money has to be stable, safe, and effective. Cryptocurrency may have some money characteristics, but it needs further research to see the characteristic of this “money.” In general, there are three groups regarding cryptocurrency: the cons, the pros, and the neutral group.

#### 2.3.1 Cons group

Bakar et al. (2017) found three conditions excluding cryptocurrency from the category of money. It is characterized by (a) no intrinsic value, (b) has an anonymous holder, and (c) it is unstable. A similar issue was proposed by Meera (2018), who suggested that “Islamic” money should be backed by an asset. Therefore, cryptocurrency does not fulfill that requirement. He suggested that to meet the Islamic principles, cryptocurrency should be backed by a real asset.

Nurhisam (2017) stated that Bitcoin is not permissible as money because it is not under government regulation and the risks and weaknesses are greater than the benefits. He was concerned with the legality of money issuance by government and uncontrollable issues.

#### 2.3.2 Pros group

Oziev and Yandiev (2018) have identified the conformity of Bitcoin to Islamic teaching and found that it has no emitter, monetary control, or transparency. Some Islamic scholars also have different opinions on this issue. The Shariah Review Bereau (2018) identifies that cryptocurrency and tokens are permissible as money as they meet habits of exchange transactions besides other requirements such as *maal* (property), *manfa'ah* (usufruct), *haqq* (right), and *dayn* (liability). Furthermore, there are some differences between coins and tokens. Tokens are also varied but the function as a medium of exchange is similar. Amalin (2018) opined that cryptocurrency fulfilled for money exchange it is transparent and clear regulation for trading. It does not contain usury (riba), which is banned in Islamic teaching. Similar reasons proposed by Zain (2018), he also stated that Bitcoin can be used for illegal transactions due to unregulated by central bank.

#### 2.3.3 Neutral group

Azulbaidi and Abdullah (2017) argue that the issue of digital money needs further study to see the suitability of Islamic teaching. This opinion was similar to that of Asif (2018), who stated that the system is not against Islamic teaching, but is not for the derivatives. Then, similar issues were raised by Bangash (2017). He stated that old Islamic scholars (such as Ibn Taymiyyah) did not specifically elaborate on the requirement of money. They only highlighted the behavior of users. Money should not be traded like a commodity, otherwise, it will cause a crisis (as stated by Imam Ibn al-Qayyim). Meanwhile, Imam Abu Hanifa and Imam Abu Yusuf permitted treating money as the commodity with some restrictions. He also concerned with the issue of safety and the unreal economy of Bitcoin.

Adam (2017) identified three requirements for money. They are (a) *māl* (wealth) (b) *taqawwum* (legal value) and (c) *thamaniyyah* (monetary usage). Bitcoin may fulfill points a and b as Bitcoin can have a value of store and is lawful based on Islamic teaching. However, it fails to be monetary usage as it has such risks as volatility, circulation, and transparency. All kinds of coins of cryptocurrency and tokens may be in line with Islamic teaching but not buy-back tokens as they must have separate contracts (Adam, 2018).

Another issue is that no more Bitcoins will be created after 2140 when 21 million Bitcoins have been created (Koropenko, 2018). Money should be able to cover any transactions as they are available for the transaction.

### 2.4 Previous research on cryptocurrency

Mills and Nower (2019) found that cryptocurrency trading is similar to gambling. However, it has low depression and anxiety. This is an experiment for gamblers who face the cryptocurrency trading experiment. They face greater problems in cryptocurrency than gambling. Antonakakis et al. (2019) stated that market uncertainty increased as the volatility of price increased as well. Akcora et al. (2018) found that the Bitcoin price was so volatile and can suffer a loss for investors.

In cryptocurrency trading, it was found that no herding activities as a trader may have an individual motive for cryptocurrency trading (Stavros &Vassilios, 2019). This may imply that there is no conspiracy in cryptocurrency trading. However, Bouri et al. (2019) found that there are other ways around it because of the increased uncertainty. Antonakakis et al. (2019) found that Bitcoin can influence the cryptocurrency market. Yi et al. (2018) also found that big cap cryptocurrency can influence volatility in the market while small cryptocurrencies have a net-transmitter volatility interconnection to others.

Ammous (2018) stated that the indicator of exchange (money) is that it is able to store a value such as Bitcoin. This is because they have a low growth supply, reliable network support protocols, and less interference. This may not be found in other cryptocurrencies that are centralized, and it is only for specific token applications.

Caporale and Zekokh (2019) criticized the use of GARCH in the single model, as it can fail to predict value-at-risk (VaR) and expected shortfall (ES) for cryptocurrency analysis, such as inefficiency risk-management, optimization of a portfolio, and derivative pricing. Platanakis and Urquhart (2019) stated that investors should be preferred in the portfolio management for cryptocurrencies by controlling estimation errors. Meanwhile, Nelson (2018) stated the impact of bubble cryptocurrency on the monetary system is small and digital money cannot replace fiat money.

This research is expected to fill the research gap by presenting the analysis based on quantitative data to justify whether cryptocurrencies meet requirements of money from the Islamic perspective. They are (a) the availability of money; money should not be limited. Money should be able to cover most transactions in the specific region. However, the supply of cryptocurrencies is limited; (b) speculation activities, money should be used for transaction not for speculation activities dominantly. This paper will show that Bitcoin was used dominantly for speculation rather than for transaction activities. Only a small portion of people would use it for a medium of exchange; and (c) stability of cryptocurrencies. This would include all cryptocurrencies in the world. Previous research only discussed specific cryptocurrencies, not all cryptocurrencies.

## 3 Research method

We apply a mixed method of descriptive literature, data study, and empirical research. In the literature study, we collected information about the ideal money from an Islamic perspective and applied it to the current context of cryptocurrency. The data were taken from a related cryptocurrency website to get updated information, while for the empirical study, the data were taken from Yahoo Finance from 29 September 2017 until 25 January 2019. Therefore, 23 cryptocurrencies can be compared, as there are new cryptocurrencies, such as Cardano (see Table 1).

**Table 1:** List of cryptocurrencies.

No.	Name	Symbol	Market Cap (in USD million at 30 September 2019)
1.	Bitcoin	BTC-USD	149,803
2.	Ethereum	ETH-USD	19,356
3.	XRP	XRP-USD	10,849
4.	Tether	USDT-USD	4,114
5.	BitcoinCash	BCH-USD	4,051
6.	Litecoin	LTC-USD	3,563
7.	EOS	EOS-USD	1.970
8.	Binance Coin	BNB-USD	2.465
9.	Stellar	XLM-USD	1.198
10	IOTA	IOT-USD	1.143
11.	UNUSSEDLEO	LEO1-USD	1.044
12.	Cardano	ADA-USD	1.021
13.	Monero	XMR-USD	961
14.	TRON	TRX-USD	957
15.	HuobiToken	HT-USD	784
16.	IOTA	MIOTA-USD	777
17.	Chainlink	LINK-USD	695
18.	Dash	DASH-USD	639
19.	Tezos	XTZ-USD	611
20.	Nano	XRB-USD	570
21.	NEO	NEO-USD	534
22.	Ethereum Classic	ETC-USD	531
23.	Cosmos	ATOM1-USD	494

We estimate the long-run volatility using the GARCH (1,1) method (Bollerslev, 1986) and we follow the implementation procedure as explained by Danielsson (2011)) as follows:

$$\sigma_{t}^{2}=\omega +\alpha y_{t-1}^{2}+\beta \sigma_{t-1}^{2}$$

(1) $$\omega =\gamma V_{LR}\;and\;\gamma +\alpha +\beta =1\;and\;\alpha +\beta <1$$

where σ_t_ and σ_t-1_ denote the volatility forecast for time t and time t-1, y_t- 1_is the realized return at time t-1, and V_LR_ is the long-run variance. This is different from Caporale and Zekokh (2019), who did not make any analysis. We also conduct robustness test to see the consistency of the test using GARCH(2,2) estimations and the structural break control.

## 4 Analysis

The analysis is based on the literature review above to qualify if the medium of exchange can be called money. Money can be in the form of any basis but it should be used for exchange and widely accessed by other people. In addition, money should be stable so the value can be maintained. The discussion is as follows:

### 4.1 The purpose of money for a medium of exchange (not only for value storage)

Money should be able to be a medium of exchange. This means that money should be used for exchange rather than for value storage. If this happens, money would be speculated to gain profit (Sanusi, 2002). This case happened in 1250 AD. Consequently, the circulation of the dinar (gold money) decreased and increased the ratio of the dinar to dirham as the number of dinars decreased as people minted dinar into gold and hoarded it (Rosly and Barakat, 2002).

**Figure 1:**
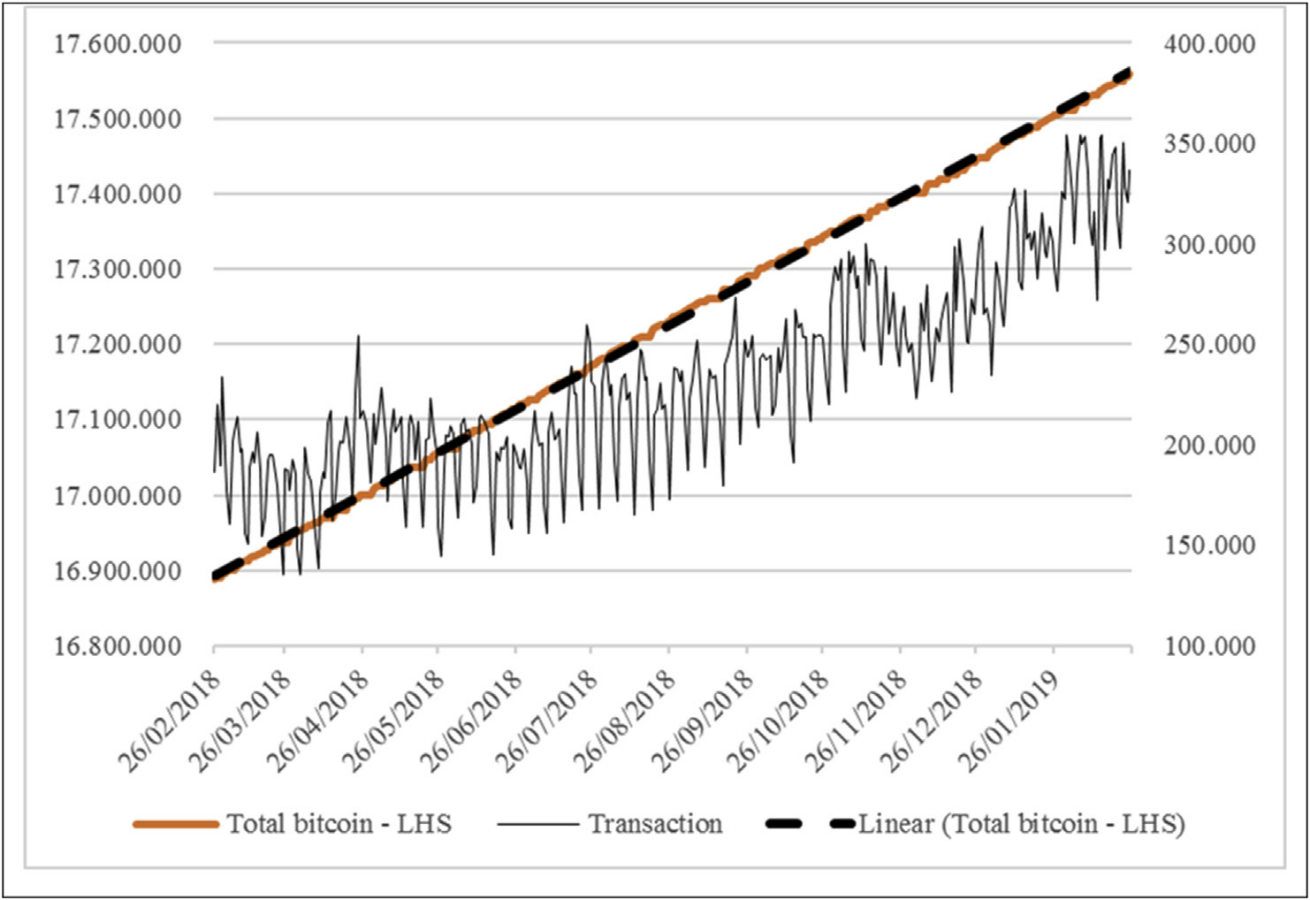
Bitcoin availability and transaction.

Source: https://www.blockchain.com

The problem is when one medium of exchange is not used for a transaction, it can increase the price because of the low supply. Therefore, demand would increase in this case. In the case of Bitcoin, in the last year, the total transaction per day was only 150.000–350.000 while the total Bitcoin increased until almost 17.600.000 units (see Figure 1). This means that only 2% of the units were used for transactions. This would hamper the situation as the total number of transactions was decreasing compared to the total Bitcoin.

**Figure 2:**
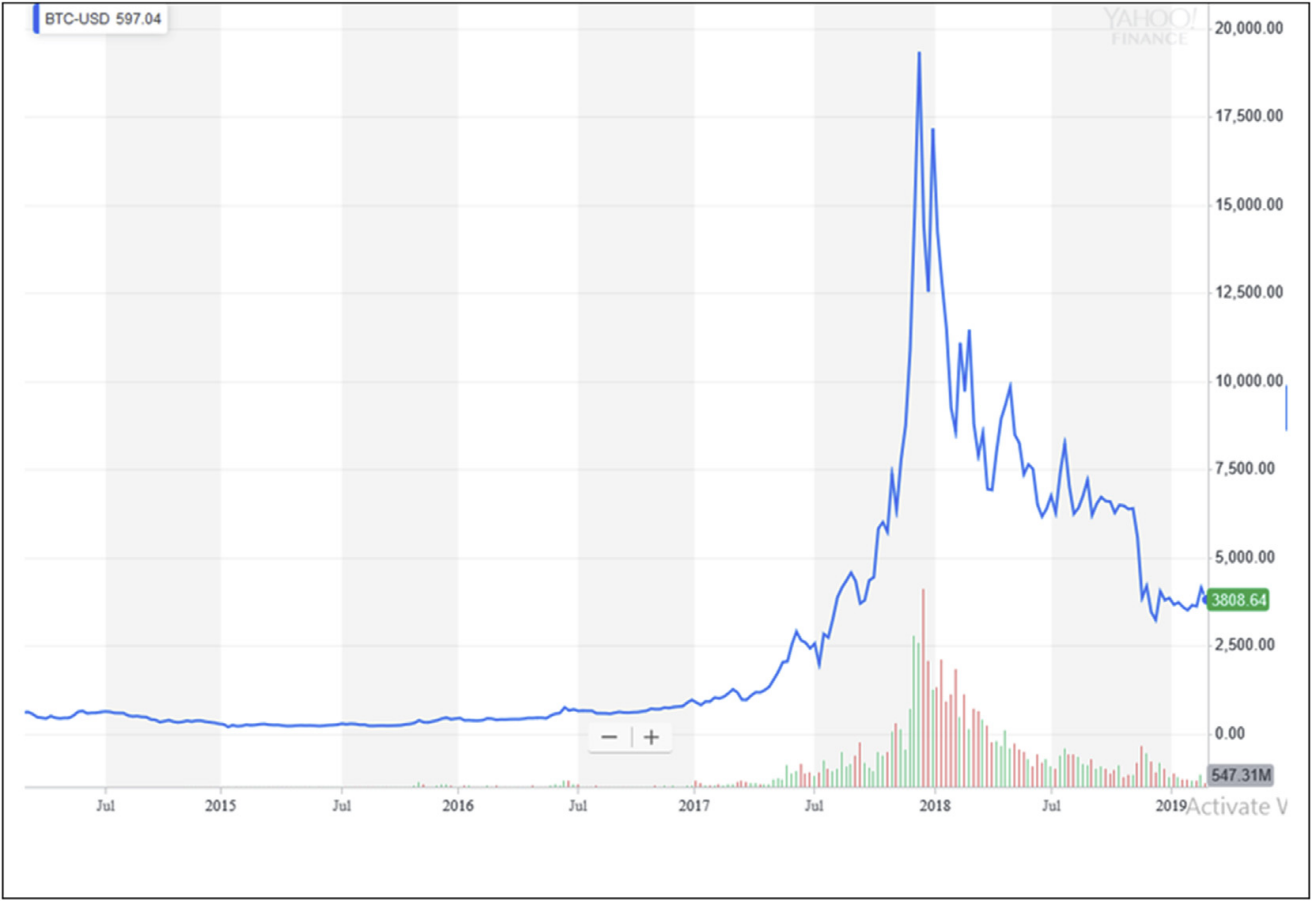
Price and volume of bitcoin trading.

Source: https://finance.yahoo.com

The next analysis is the use of Bitcoin for speculation. In Figure 2, we can see that the number of volume trading increased when the price also increased until the top. Then, when the price started to decrease, the volume of the transaction is also quite high. The volume decreased as the price decreased. The supply and demand for Bitcoin transactions may be small in terms of the volume of bid and offer. Consequently, the price is easily volatile.

**Figure 3:**
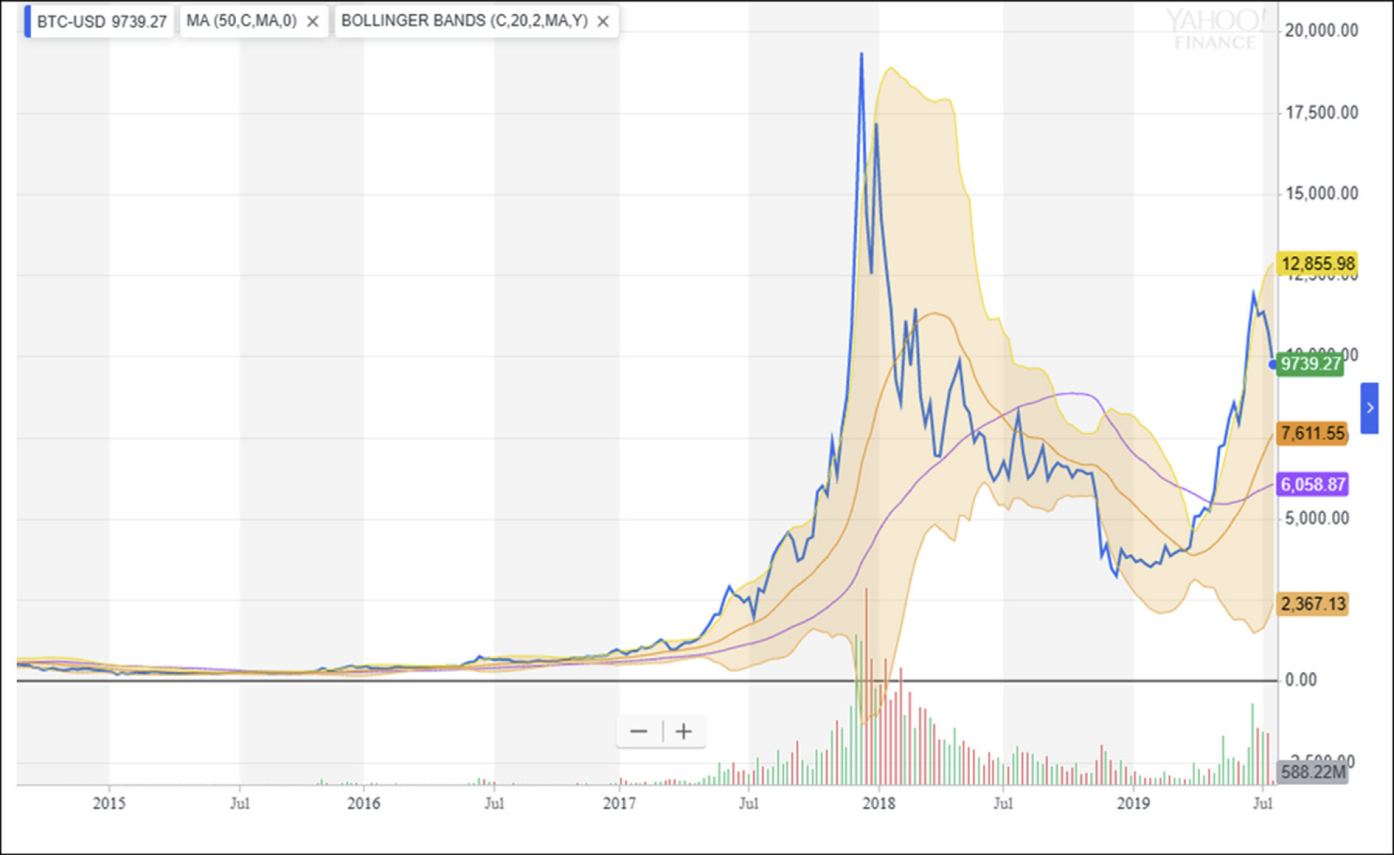
Technical analysis of bitcoin.

Source: https://finance.yahoo.com

In this case, technical analysis, which is usually used for stock price prediction, may be useless because of the high volatility. Using moving average 50 (MA 50) and Bollinger bands may be useful if the movement is not so volatile and has some pattern (see Figure 3). There needs to be further research to predict the movement of Bitcoin. This, however, may support Mills and Nower (2019) who classify trading cryptocurrency as similar to gambling, which cannot be predicted accurately.

### 4.2 Cryptocurrency supply

It is stated that the Bitcoin supply is only up to 21 million units. Therefore, this can be an interesting issue that not all transactions can be covered by Bitcoin.^1^ Ammous (2018) found that Bitcoin is the ideal money in this case. However, other cryptocurrencies may be backed up for this issue. However, cryptocurrency consists of two types: token and coin. The difference is that the token depends on another cryptocurrency as a platform to operate.^2^ Some platforms are Ethereum (biggest), NEM, and Omni.^3^ A token is like a contract to Bitcoin, which is then further developed into types such as the work token, utility token, asset-backed token, revenue token, equity token, and buy-back token. The user of a token has the right of the application (Adam, 2018).

In Tables 2 and 3, we can see the maximum supply for each cryptocurrency. Some cryptocurrencies like Ethereum, EOS, Tether and Stellar are not available for maximum supply information. This is also an important issue that should be raised if the supply of “money” is limited. However, other substitutes or other similar complementary money can solve this issue.

**Table 2:** Token of cryptocurrency.

Name	Platform	Market Cap (USD)	Circulating Supply	Maximum Supply
Kin	Ethereum	18,595,854	756,097,560,976	-
Dentacoin	Ethereum	18,052,031	326,822,211,298	8,000,000,000,000
Pundi X	Ethereum	115,100,605	170,357,512,833	-
Holo	Ethereum	153,006,464	133,214,575,156	-
Dent	Ethereum	28,741,189	32,456,475,076	-
IOST	Ethereum	87,096,759	12,013,965,609	-
Zilliqa	Ethereum	145,488,729	8,279,187,391	-
Cube	Ethereum	16,329,944	6,774,940,000	-

**Table 3:** Coin of cryptocurrency.

Name	Market Cap (USD)	Circulating Supply	Maximum Supply
Bitcoin	67,479,674,708	17,560,300	21,000,000
Ethereum	14,373,564,267	105,023,360	-
XRP	13,048,666,897	41,365,634,610	100,000,000,000
EOS	3,119,855,774	906,245,118	-
Litecoin	2,734,912,520	60,649,561	84,000,000
Bitcoin Cash	2,327,677,561	17,644,088	21,000,000
Tether	2,039,780,527	2,021,459,017	-
Stellar	1,658,606,724	19,205,488,369	-

### 4.3 Stability of monetary value

The next important issue for money is stability. This issue is required by the Accounting and Auditing Organization for Islamic Financial Institution (AAOIFI) in the accounting assumption. Monetary stability is one of the assumptions in accounting; otherwise, it cannot be useful for presenting the financial information. Using GARCH (1,1) we can see the stability of cryptocurrency. Refereeing to the model, ω, α and β are the parameters to be estimated. Likewise, once we obtain ω, α, and β parameters, we can easily calculate γ and the long-run variance (V_LR_). Note that the long-run volatility (σ_LR_) can be obtained by square rooting the long-run variance. Table 4 reports the parameters, long-run variance, long-run volatility and relative long run (LR) risk estimates of the 23 cryptocurrencies and S&P500. The relative LR risk is the cryptocurrency's volatility divided by the S&P500's volatility. Overall, we can see that almost all of the cryptocurrencies have a substantially higher risk than that of S&P500. Only USDT cryptocurrency has lower risk than that of S&P500. Most of the cryptocurrencies have around three or four times the risk than that of S&P500. Even DOGE has more thirteen times risk than that of S&P500. This indicates that cryptocurrencies tend to be unstable. These results are consistent with Antonakakis et al. (2019) and Akcora et al. (2018) findings.

**Table 4:** The parameters, long-run variance, long-run volatility and relative long run (LR) risk GARCH(1,1) estimates of the 23 cryptocurrencies and S&P500.

Parameters	TRX	BTC	XRP	ETH	LTC	BCH
Ω	0.000	0.000	0.000	0.000	0.000	0.000
Α	0.046	0.059	0.178	0.076	0.098	0.115
Β	0.949	0.925	0.793	0.852	0.859	0.833
Γ	0.004	0.016	0.028	0.071	0.042	0.053
V_LR_	0.011	0.002	0.010	0.003	0.004	0.008
σ_LR_	0.103	0.045	0.099	0.058	0.065	0.091
Relative LR Risk	4.675	2.036	4.505	2.629	2.949	4.165
Parameters	USDT	EOS	BNB	XLM	ADA	XMR
Ω	0.000	0.000	0.000	0.000	0.000	0.000
Α	1.000	0.032	0.128	0.068	0.215	0.059
Β	0.451	0.962	0.859	0.930	0.792	0.911
Γ	0.451	0.006	0.013	0.002	0.007	0.030
V_LR_	0.000	0.007	0.009	0.021	0.046	0.004
σ_LR_	0.004	0.086	0.094	0.146	0.215	0.066
Relative LR Risk	0.173	3.920	4.292	6.642	9.791	2.993
Parameters	IOT	DASH	NEO	LINK	XEM	ZEC
Ω	0.000	0.000	0.000	0.000	0.001	0.000
Α	0.095	0.125	0.061	0.046	1.000	0.070
Β	0.864	0.829	0.908	0.942	0.212	0.895
Γ	0.041	0.046	0.031	0.011	0.212	0.034
V_LR_	0.007	0.006	0.006	0.008	0.006	0.004
σ_LR_	0.082	0.076	0.077	0.091	0.078	0.064
Relative LR Risk	3.750	3.463	3.525	4.124	3.545	2.927
Parameters	WAVES	ZRX	DOGE	QTUM	BAT	S&P500
Ω	0.000	0.000	0.000	0.001	0.000	0.000
Α	0.282	0.045	0.247	0.609	0.040	0.261
Β	0.670	0.934	0.757	0.512	0.932	0.745
Γ	0.048	0.021	0.003	0.121	0.028	0.006
V_LR_	0.009	0.007	0.083	0.012	0.006	0.000
σ_LR_	0.094	0.082	0.288	0.111	0.079	0.022
Relative LR Risk	4.293	3.755	13.122	5.031	3.578	1.000

We also performed another estimation for GARCH(2,2). Table 5 reports the parameters, long-run variance, long-run volatility and relative long-run (LR) risk GARCH(2,2) estimates of the 23 cryptocurrencies and S&P500. Again, we can see that almost all of the cryptocurrencies have substantially higher risk than that of S&P500. Only USDT cryptocurrency has a lower risk than that of S&P500. Most of the cryptocurrencies have around four or five times of risk than that of S&P500, even DOGE has more twenty-three times risk than that of S&P500. Overall, cryptocurrencies tend to be unstable.

**Table 5:** The parameters, long run variance, long run volatility and relative long run (LR) risk GARCH(2,2) estimates of the 23 cryptocurrencies and S&P500.

Parameters	TRX	BTC	XRP	ETH	LTC	BCH
ω	0.000	0.000	0.000	0.000	0.000	0.001
α1	0.066	0.097	0.127	0.110	0.139	0.145
α2	0.498	0.140	0.047	0.232	0.017	0.000
β1	0.000	0.000	0.173	0.000	0.031	0.020
β2	0.430	0.739	0.596	0.557	0.734	0.749
γ	0.007	0.024	0.056	0.101	0.079	0.085
VLR	0.010	0.002	0.009	0.003	0.004	0.008
σLR	0.100	0.044	0.094	0.058	0.064	0.089
Relative LR Risk	5.451	2.420	5.101	3.137	3.495	4.853
Parameters	USDT	EOS	BNB	XLM	ADA	XMR
ω	0.000	0.000	0.000	0.000	0.000	0.000
α1	0.167	0.000	0.140	0.113	0.278	0.000
α2	0.270	0.955	0.465	0.288	0.273	0.694
β1	0.000	0.038	0.000	0.000	0.000	0.103
β2	0.612	0.000	0.374	0.596	0.447	0.156
γ	0.049	0.007	0.021	0.003	0.002	0.047
VLR	0.000	0.008	0.006	0.024	0.184	0.004
σLR	0.002	0.089	0.080	0.156	0.428	0.067
Relative LR Risk	0.092	4.864	4.377	8.524	23.368	3.631
Parameters	IOT	DASH	NEO	LINK	XEM	ZEC
ω	0.000	0.000	0.000	0.000	0.001	0.000
α1	0.136	0.232	0.011	0.087	1.000	0.077
α2	0.309	0.153	0.890	0.182	0.157	0.335
β1	0.000	0.000	0.069	0.000	0.000	0.032
β2	0.492	0.561	0.000	0.711	0.041	0.503
γ	0.063	0.054	0.030	0.021	0.198	0.053
VLR	0.007	0.007	0.006	0.008	0.007	0.004
σLR	0.081	0.085	0.080	0.091	0.081	0.064
Relative LR Risk	4.428	4.648	4.351	4.944	4.439	3.505
Parameters	WAVES	ZRX	DOGE	QTUM	BAT	S&P500
ω	0.000	0.000	0.000	0.002	0.000	0.000
α1	0.191	0.000	0.316	0.396	0.056	0.219
α2	0.618	0.928	0.260	0.444	0.482	0.000
β1	0.143	0.051	0.000	0.225	0.000	0.245
β2	0.000	0.000	0.426	0.000	0.420	0.551
γ	0.048	0.021	0.003	0.065	0.041	0.015
VLR	0.010	0.007	0.104	0.029	0.006	0.000
σLR	0.099	0.083	0.323	0.170	0.078	0.018
Relative LR Risk	5.425	4.525	17.621	9.258	4.280	1.000

We also performed GARCH(1,1) and GARCH(2,2) estimations with the structural break control. We selected December 7, 2017 as the point of structural break because that is the day when BTC experienced the highest absolute return. Tables 6 and 7 report the parameters, long-run variance, long-run volatility and relative long-run (LR) risk GARCH(1,1) estimates of the 23 cryptocurrencies and S&P500 during the period before and after the structural break. Tables 8 and 9 report the parameters, long-run variance, long-run volatility and relative long-run (LR) risk GARCH(2,2) estimates of the 23 cryptocurrencies and S&P500 during the period before and after the structural break. We denote “EXPL” for some estimations producing explosive GARCH. An explosive GARCH provides an infinity estimation of the long-run variance (Zivot, 2008). We obtain consistent results. Only one cryptocurrency reports a lower relative risk for GARCH(1,1) estimate during the period before the structural break, seven cryptocurrencies report the lower relative risk for GARCH(2,2) estimate during the period before the structural break, and one cryptocurrency reports the lower relative risk for GARCH(2,2) estimate during the period after the structural break. Overall, we conclude that cryptocurrencies tend to be unstable.

**Table 6:** The parameters, long run variance, long run volatility and relative long run (LR) risk GARCH(1,1) estimates of the 23 cryptocurrencies and S&P500 for observations before December 7, 2017.

Parameters	TRX	BTC	XRP	ETH	LTC	BCH
ω	0.012	0.002	0.002	EXPL	EXPL	0.002
α	0.000	0.303	0.195			0.049
β	0.654	0.000	0.000			0.778
γ	0.346	0.697	0.805			0.173
VLR	0.035	0.003	0.003			0.013
σLR	0.186	0.050	0.052			0.115
Relative LR Risk	54.076	14.546	15.176			33.451
Parameters	USDT	EOS	BNB	XLM	ADA	XMR
ω	0.000	EXPL	0.001	0.012	EXPL	0.000
α	0.000		0.223	0.036		0.101
β	0.953		0.692	0.521		0.845
γ	0.047		0.085	0.443		0.054
VLR	0.000		0.006	0.026		0.005
σLR	0.009		0.078	0.163		0.071
Relative LR Risk	2.726		22.757	47.279		20.690
Parameters	IOT	DASH	NEO	LINK	XEM	ZEC
ω	0.000	0.005	EXPL	EXPL	0.001	0.000
α	0.177	0.054			0.493	0.057
β	0.871	0.000			0.099	0.914
γ	0.049	0.946			0.408	0.028
VLR	0.004	0.005			0.004	0.004
σLR	0.067	0.069			0.059	0.059
Relative LR Risk	19.393	20.108			17.281	17.262
Parameters	WAVES	ZRX	DOGE	QTUM	BAT	S&P500
ω	0.001	EXPL	EXPL	EXPL	0.000	0.000
α	0.237				0.005	0.067
β	0.657				1.000	0.671
γ	0.106				0.005	0.262
VLR	0.007				0.000	0.000
σLR	0.086				0.001	0.003
Relative LR Risk	25.090				0.319	1.000

**Table 7:** The parameters, long run variance, long run volatility and relative long run (LR) risk GARCH(1,1) estimates of the 23 cryptocurrencies and S&P500 for observations after December 7, 2017.

Parameters	TRX	BTC	XRP	ETH	LTC	BCH
ω	0.001	0.000	0.000	0.000	0.000	0.001
α	0.322	0.061	0.144	0.066	0.117	0.171
β	0.623	0.918	0.827	0.867	0.828	0.700
γ	0.055	0.021	0.029	0.066	0.055	0.129
VLR	0.015	0.002	0.007	0.003	0.004	0.006
σLR	0.122	0.040	0.086	0.058	0.060	0.080
Relative LR Risk	8.877	2.941	6.263	4.220	4.381	5.850
Parameters	USDT	EOS	BNB	XLM	ADA	XMR
ω	0.000	0.000	0.000	0.000	0.000	0.000
α	1.000	0.028	0.090	0.078	0.078	0.049
β	0.261	0.964	0.895	0.900	0.890	0.924
γ	0.261	0.008	0.015	0.022	0.032	0.027
VLR	0.000	0.005	0.006	0.005	0.006	0.004
σLR	0.006	0.071	0.074	0.071	0.078	0.062
Relative LR Risk	0.434	5.164	5.414	5.175	5.692	4.498
Parameters	IOT	DASH	NEO	LINK	XEM	ZEC
ω	0.000	0.000	0.000	0.000	0.000	0.000
α	0.069	0.091	0.084	0.052	0.010	0.060
β	0.881	0.877	0.884	0.932	0.971	0.909
γ	0.049	0.032	0.032	0.016	0.019	0.031
VLR	0.005	0.004	0.006	0.007	0.002	0.004
σLR	0.072	0.067	0.074	0.086	0.044	0.062
Relative LR Risk	5.236	4.843	5.408	6.224	3.201	4.544
Parameters	WAVES	ZRX	DOGE	QTUM	BAT	S&P500
ω	0.000	0.000	0.000	0.001	0.000	0.000
α	0.276	0.029	0.433	0.427	0.039	0.228
β	0.670	0.959	0.628	0.589	0.932	0.742
γ	0.054	0.012	0.061	0.016	0.029	0.030
VLR	0.008	0.005	0.005	0.060	0.006	0.000
σLR	0.088	0.072	0.068	0.244	0.075	0.014
Relative LR Risk	6.389	5.219	4.977	17.779	5.471	1.000

**Table 8:** The parameters, long run variance, long run volatility and relative long run (LR) risk GARCH(2,2) estimates of the 23 cryptocurrencies and S&P500 for observations before December 7, 2017.

Parameters	TRX	BTC	XRP	ETH	LTC	BCH
ω	0.016	0.001	0.001	0.000	0.000	0.003
α1	0.000	0.289	0.225	0.000	0.000	0.087
α2	0.066	0.000	0.000	1.000	1.000	0.000
β1	0.000	0.000	0.000	0.000	0.000	0.000
β2	0.473	0.154	0.261	0.004	0.002	0.719
γ	0.462	0.557	0.514	0.004	0.002	0.193
VLR	0.035	0.003	0.003	0.000	0.000	0.013
σLR	0.186	0.051	0.053	0.001	0.001	0.116
Relative LR Risk	54.754	14.858	15.460	0.180	0.307	34.068
Parameters	USDT	EOS	BNB	XLM	ADA	XMR
ω	0.000	0.001	0.001	0.021	0.004	0.000
α1	0.072	0.000	0.116	0.023	1.000	0.044
α2	0.000	0.817	0.000	0.000	0.025	0.000
β1	0.000	0.115	0.244	0.037	0.000	0.145
β2	0.473	0.000	0.489	0.156	0.000	0.783
γ	0.454	0.068	0.151	0.784	0.025	0.027
VLR	0.000	0.013	0.006	0.026	0.170	0.010
σLR	0.009	0.116	0.078	0.162	0.412	0.099
Relative LR Risk	2.623	33.985	22.810	47.633	121.226	28.969
Parameters	IOT	DASH	NEO	LINK	XEM	ZEC
ω	0.001	0.005	0.000	0.000	0.002	0.000
α1	0.239	0.053	0.000	0.000	0.493	0.000
α2	0.278	0.000	1.000	1.000	0.000	0.393
β1	0.000	0.000	0.000	0.000	0.077	0.133
β2	0.508	0.000	0.004	0.003	0.000	0.380
γ	0.025	0.947	0.004	0.003	0.430	0.094
VLR	0.026	0.005	0.000	0.000	0.004	0.003
σLR	0.161	0.069	0.001	0.002	0.060	0.054
Relative LR Risk	47.266	20.352	0.352	0.583	17.557	15.845
Parameters	WAVES	ZRX	DOGE	QTUM	BAT	S&P500
ω	0.001	0.000	0.000	0.006	0.000	0.000
α1	0.037	0.000	0.000	0.000	0.000	0.011
α2	0.578	1.000	1.000	0.000	1.000	0.000
β1	0.232	0.000	0.000	0.021	0.000	0.151
β2	0.000	0.003	0.005	0.666	0.004	0.258
γ	0.153	0.003	0.005	0.313	0.004	0.580
VLR	0.007	0.000	0.000	0.019	0.000	0.000
σLR	0.083	0.001	0.001	0.139	0.001	0.003
Relative LR Risk	24.398	0.380	0.287	41.010	0.361	1.000

**Table 9:** The parameters, long run variance, long run volatility and relative long run (LR) risk GARCH(2,2) estimates of the 23 cryptocurrencies and S&P500 for observations after December 7, 2017.

Parameters	TRX	BTC	XRP	ETH	LTC	BCH
ω	0.001	0.000	0.000	0.000	0.000	0.001
α1	0.325	0.018	0.095	0.097	0.069	0.216
α2	0.406	0.908	0.056	0.243	0.694	0.304
β1	0.000	0.051	0.159	0.000	0.082	0.000
β2	0.210	0.000	0.637	0.562	0.084	0.304
γ	0.058	0.023	0.054	0.098	0.070	0.176
VLR	0.013	0.002	0.007	0.003	0.004	0.006
σLR	0.115	0.041	0.085	0.058	0.062	0.080
Relative LR Risk	8.910	3.143	6.604	4.487	4.788	6.198
Parameters	USDT	EOS	BNB	XLM	ADA	XMR
ω	0.000	0.000	0.000	0.000	0.000	0.000
α1	0.188	0.000	0.114	0.015	0.004	0.000
α2	0.279	0.171	0.450	0.892	0.014	0.752
β1	0.000	0.071	0.000	0.066	0.168	0.093
β2	0.594	0.736	0.414	0.000	0.775	0.109
γ	0.060	0.022	0.022	0.027	0.040	0.046
VLR	0.000	0.006	0.005	0.005	0.008	0.004
σLR	0.002	0.077	0.070	0.070	0.090	0.064
Relative LR Risk	0.127	5.986	5.390	5.405	6.975	4.938
Parameters	IOT	DASH	NEO	LINK	XEM	ZEC
ω	0.000	0.000	0.000	0.000	0.000	0.000
α1	0.090	0.012	0.034	0.102	0.000	0.077
α2	0.197	0.858	0.866	0.169	0.974	0.382
β1	0.037	0.095	0.067	0.000	0.008	0.012
β2	0.589	0.000	0.000	0.700	0.000	0.483
γ	0.087	0.035	0.033	0.029	0.018	0.046
VLR	0.005	0.004	0.006	0.008	0.002	0.004
σLR	0.073	0.067	0.076	0.087	0.044	0.063
Relative LR Risk	5.662	5.169	5.868	6.721	3.377	4.847
Parameters	WAVES	ZRX	DOGE	QTUM	BAT	S&P500
ω	0.000	0.000	0.000	0.002	0.000	0.000
α1	0.205	0.008	0.472	0.114	0.053	0.268
α2	0.624	0.954	0.357	0.127	0.527	0.324
β1	0.116	0.025	0.000	0.558	0.000	0.030
β2	0.000	0.000	0.221	0.122	0.379	0.332
γ	0.055	0.013	0.050	0.078	0.041	0.046
VLR	0.008	0.005	0.006	0.026	0.006	0.000
σLR	0.091	0.072	0.080	0.161	0.075	0.013
Relative LR Risk	7.061	5.597	6.230	12.472	5.824	1.000

## 5 Conclusion

The discourse of cryptocurrency as money or otherwise is important as this will be useful for Muslims, as a big part of the world population. In big countries like China and Russia, cryptocurrency is banned as a result of reasons such as safety and risks. In Muslim countries, which are bound to Islamic teaching, the permissibility of cryptocurrency is an important issue in the Islamic perspective. So far, no single Muslim country permits cryptocurrency for legal transactions. While it is quite difficult to identify the purpose of using specific money, in the case of cryptocurrency, such as Bitcoin, most users have a speculative motive to gaining profit instead of for exchange as with money. This is interesting, as the characteristic of cryptocurrency may not be liquid and stable for conversion. In the case of supply, not all cryptocurrencies have a sufficient supply to cover all transactions. In addition, this must be supported by available online merchants, especially for electronic transactions. The last issue is stability. Cryptocurrency is more volatile than the S&P itself. This result is also consistent with robustness tests. Users must be aware of these characteristics. Therefore, cryptocurrency may be classified as a medium of exchange of digital currency rather than money for this category. Money has flexible characteristics from cryptocurrency and its stability can be intervened by the government.

## Declarations

### Author contribution statement

Dodik Siswantoro, Rangga Handika, Aria Farah Mita: Conceived and designed the experiments; Performed the experiments; Analyzed and interpreted the data; Contributed reagents, materials, analysis tools or data; Wrote the paper.

### Funding statement

This work was supported by Universitas Indonesia under grant Q1Q2.

### Competing interest statement

The authors declare no conflict of interest.

### Additional information

No additional information is available for this paper.

## Footnotes

1 https://en.bitcoin.it/wiki/Controlled_supply.

2 https://coinmarketcap.com/faq/.

3 https://coinmarketcap.com/tokens/.
